# Comparative Evaluation of Microhardness by Common Drinks on Esthetic Restorative Materials and Enamel: An *in vitro* Study

**DOI:** 10.5005/jp-journals-10005-1503

**Published:** 2018-06-01

**Authors:** Roli Gupta, Manish Madan, Parminder Dua, Sheeba Saini, Ritu Mangla, Trilok Kainthla, Akash Dupper

**Affiliations:** 1 Postgraduate Student, Department of Pedodontics and Preventive Dentistry, Himachal Institute of Dental Sciences and Hospital, Paonta Sahib, Himachal Pradesh India; 2 Professor and Head, Department of Pedodontics and Preventive Dentistry, Himachal Institute of Dental Sciences and Hospital, Paonta Sahib, Himachal Pradesh India; 3 Professor, Department of Pedodontics and Preventive Dentistry, Himachal Institute of Dental Sciences and Hospital, Paonta Sahib, Himachal Pradesh India; 4 Reader, Department of Pedodontics and Preventive Dentistry, Himachal Institute of Dental Sciences and Hospital, Paonta Sahib, Himachal Pradesh India; 5 Senior Lecturer, Department of Pedodontics and Preventive Dentistry, Himachal Institute of Dental Sciences and Hospital, Paonta Sahib, Himachal Pradesh India; 6 Postgraduate Student, Department of Conservative Dentistry and Endodontics Himachal Institute of Dental Sciences and Hospital, Paonta Sahib, Himachal Pradesh, India; 7 Reader, Department of Conservative Dentistry and Endodontics Yamuna Institute of Dental Sciences and Research, Gadholi Haryana, India

**Keywords:** Beverages, Composite resins, Microhardness.

## Abstract

**Aim:**

This study was aimed to evaluate effects of various beverages on microhardness of esthetic restorative materials.

**Materials and methods:**

A total of 160 disk-like specimens were prepared with 40 specimens each using nanocomposite resin, nano-ionomer, compomer, and conventional composite resin as experimental groups. Forty primary teeth were prepared and mounted in acrylic to be used as control group. Microhardness of the restorative materials was measured using Vickers microhardness tester at baseline and after immersion in various beverages. The difference between the two readings was evaluated within different groups.

**Results:**

In general, low pH beverages adversely affected the properties of the tested materials. Microhardness of tested materials was significantly decreased after immersion in various beverages with the exception of Yakult. After the immersion period, the enamel showed the maximum loss in microhardness followed by nano-ionomer.

**Conclusion:**

Low pH beverages were the most aggressive media for enamel, nano-ionomer and compomer, but in contrast, composite resin was relatively less affected. Probiotic drink appeared relatively benign toward the tested materials.

**How to cite this article:** Gupta R, Madan M, Dua P, Saini S, Mangla R, Kainthla T, Dupper A. Comparative Evaluation of Microhardness by Common Drinks on Esthetic Restorative Materials and Enamel: An *in vitro* Study. Int J Clin Pediatr Dent 2018;11(3):155-160.

## INTRODUCTION

Dental erosion is the result of a pathologic, chronic, localized loss of dental hard tissue that is chemically etched away from the tooth surface by acid and/or chelation without bacterial involvement.^1^ This process may be caused by either extrinsic or intrinsic agents.^2^ The intrinsic causes comprise recurrent vomiting as in patients suffering from anorexia and bulimia, cytostatic drug treatment, or propulsion of gastric contents into the mouth due to gastroesophageal reflux. Extrinsic causes comprise frequent consumption of acidic foods or drinks, the use of acidic hygiene products and acidic medicines, such as effervescent vitamin C or aspirin.^3^ The consumption of soft drinks, sport drinks, and acid juices can decrease the pH of oral environment below the critical pH of 5.5 and subsequently lead to enamel and dentin demineraliza-tion.^4^ Currently, dental erosion is considered a significant clinical problem in the oral health of schoolchildren and young adults.^5^

A number of restorative materials are currently available to replace natural tooth, and demand for products with good mechanical and caries-protective properties, together with a simple clinical application procedure, has led to the development of glass-ionomer cement, resin-modified glass-ionomer cement, compomer, and resin composite.^6^ Ever since the introduction of glass-ionomer cements to the dental profession by Wilson and Kent in 1972,^7^ they have undergone a lot of modifications with time. They have been combined with composites to form polyacid-modified resin composites, or “compomers.” Development in the field of resin-modified glass-ionomer cement has led to the introduction of nano-ionomer, which combines the benefit of resin-modified glass-ionomer cement together with nanofiller technology.^8^

The chemical environment is one aspect of the oral environment, which could have an appreciable influence on the *in vivo* degradation of restorations due to which the load resistance of materials decreases (microhardness) at clinically detectable level.^9^ The present study was therefore, carried out in order to provide information on microhardness and to provide data on the relative abilities of a range of contemporary restorative materials to resist attack by these beverages.

## MATERIALS AND METHODS

The present study was carried out in the Department of Pedodontics and Preventive Dentistry, Himachal Institute of Dental Sciences & Hospital, Paonta Sahib, Himachal Pradesh, India, to evaluate the erosive potential of commercially available drinks, i.e., aerated drink Coca-Cola, orange juice (Minute Maid), lemon juice (Rasna), fermented milk (Yakult) on the surface of tooth enamel and different tooth-colored restorative materials.

The pH of the test solutions was determined using a calibrated pH meter (accurate pH electrode) which was calibrated using test solutions of standard buffer of pH 9.20 and checked again at pH 4.0. Each drink from freshly opened bottle was placed in a glass beaker. Each sample was brought down to room temperature before reading its pH. The measure of pH was found to be 1.36 for Coca-Cola, 3.43 for Pulpy Orange, 3.46 for Rasna, and 3.69 for Yakult.

### Restorative Materials and Beverages used in the Study

- Conventional hybrid composite, Tetric-Econom (Ivoclar Vivadent)
- Nanohybrid composite, Ceram-X (Dentsply, Germany)
- Compomer, Compoglas F (Ivoclar, Vivadent)
- Nano-ionomer, Ketac N 100 (3M, ESPE)
- Aerated carbonated drink, Coca-Cola
- Orange juice, Pulpy Orange (Minute Maid)
- Lemon juice, Nimboo Pani (Rasna)
- Fermented milk, Yakult (Yakult, India)

### Preparation of Control Group

The teeth were cleaned thoroughly and de-coronated using diamond disk and low-speed handpiece (NSK, Japan). Placing buccal portions at the top, the crowns were embedded in clear acrylic resin blocks with the outer enamel surface exposed and were polished using abrasive grits of 600, 800, 1000, 1200, and 1500 (3M)

### Preparation of Experimental Groups

- Preparation of stainless steel mold: A stainless steel mold was constructed of 8 mm diameter and 2 mm thickness dimension in circular shape to prepare the specimens of experimental materials.

Preparation of specimens: Forty disk-shaped specimens of each restorative material with a total of 160 were prepared using a stainless steel mold of dimension 8 (diameter) × 2 (thickness) mm. The stainless steel mold was placed on glass slab and restorative material placed in it. Over the top, a cover slip was placed to squeeze out excess material and cured for 40 seconds. After curing from the top, curing was done from the bottom of glass slab for 40 seconds and specimen was taken out of the mold.

### Grouping of Samples

The prepared 200 specimens (tooth and restorative materials) were divided into five groups, each containing 40 samples:

Group I—Conventional hybrid composite

Group II—Nanohybrid composite

Group III—Compomer

Group IV—Nano-ionomer

Group V—Tooth enamel (control)

Each group was further subdivided randomly into four subgroups of 10 teeth each depending on the testing media (experimental drinks) (n = 10) whose erosive potential was to be evaluated. The various groups and subgroups were:

- Subgroup A I (n = 10): Conventional hybrid composite immersed in Coca-Cola
- Subgroup A II (n = 10) : Conventional hybrid composite immersed in Pulpy Orange
- Subgroup A III (n = 10) : Conventional hybrid composite immersed in Yakult
- Subgroup A IV (n = 10) : Conventional hybrid composite immersed in Rasna
- Subgroup B I (n = 10): Nanocomposite immersed in Coca-Cola
- Subgroup B II (n = 10): Nanocomposite immersed in Pulpy Orange
- Subgroup B III (n = 10): Nanocomposite immersed in Yakult
- Subgroup B IV (n = 10): Nanocomposite immersed in Rasna
- Subgroup C I (n = 10): Compomer immersed in Coca-Cola
- Subgroup C II (n = 10): Compomer immersed in Pulpy Orange
- Subgroup C III (n = 10): Compomer immersed in Yakult
- Subgroup C IV (n = 10): Compomer immersed in Rasna
- Subgroup D I (n = 10): Nano-ionomer immersed in Coca-Cola
- Subgroup D II (n = 10): Nano-ionomer immersed in Pulpy Orange
- Subgroup D III (n = 10): Nano-ionomer immersed in Yakult
- Subgroup D IV (n = 10): Nano-ionomer immersed in Rasna
- Subgroup E I (n = 10): Extracted deciduous teeth immersed in Coca-Cola
- Subgroup E II (n = 10): Extracted deciduous teeth immersed in Pulpy Orange
- Subgroup E III (n = 10): Extracted deciduous teeth immersed in Yakult
- Subgroup E IV (n = 10): Extracted deciduous teeth immersed in Rasna.

**Table 1:** **Table 1:** Change in hardness (AVHN) of the restorative materials in various immersion media

		*Conventional hybrid*		*Nanocomposite*		*Compomer*		*Nano-ionomer*		*Enamel*	
Coke		–41.1 ± 14.9^a^		–69.7 ± 30.6		–94.4 ± 39.4		–126.5 ± 43.1^b^		–176.7 ± 52.7^c^	
Pulpy Orange		–31.3 ± 11.6		–65 ± 51.5		–90.7 ± 166.3		–96.3 ± 33.5		–154 ± 45.8	
Yakult		–27.8 ± 8.2^a^		–40.6 ± 10.4		–82.3 ± 14.8		–85.7 ± 17.7 ^b^		–121.8 ± 40.7^c^	
Rasna		–32.4 ± 3.1		–64.4 ± 11.3		–92.3 ± 34.4		–95.3 ± 20.2		–137.2 ± 41.5	
Total		–33.2 ± 11.1		–59.9 ± 31.9		–90 ± 84.2		–100.9 ± 33.1		–147.4 ± 48.3	

a, b, c—difference is significant between the same alphabet groups; (–) sign depicts decrease in microhardness

These samples were stored in saline until the immersion regimen began.

### Surface Hardness Testing

The samples were blotted dry using tissue paper and the baseline readings were obtained for surface hardness. VHM 002V (Walter UHL, Germany) was used to measure the microhardness of each sample before and after the immersion regimen in testing drinks. To record the reading, a force of 10 gm for 15 seconds was applied on the exposed surface of specimen following the protocol testing by Yanikoğlu et al.^10^ Three consecutive readings were made and their arithmetic mean was taken as baseline (Vickers hardness number, VHN_1_).

After baseline readings, the samples were immersed in the respective beverages. The immersion regimen followed was as follows: The samples in each group were immersed in the respective beverage for 10 minutes every day. For the remaining part of the day, the samples were kept immersed in distilled water. This regimen was followed for 56 days.

At the end of the test period/immersion period, the average microhardness of three readings (VHN_2_) of each sample was evaluated in a way similar to that done for baseline surface hardness evaluation. The postimmersion values were tabulated as final microhardness values for each specimen.

### Change in Microhardness

The difference in microhardness (AVHN) was calculated by using the equation similar to other microhardness test (KHN).^11^

ΔVHN= VHN_2_ - VHN_1:_

where VHN_1_ is the preconditioning value and VHN_2_ is the microhardness after immersion regimen.

### Statistical Analysis

One-way analysis of variance (ANOVA) and *post hoc* Tukey test were used to compare the microhardness values among the four restorative materials and enamel, among four different immersion solutions (Coca-Cola, Pulpy Orange, Rasna, and Yakult), and between two different immersion times (baseline and 56 days).

## RESULTS

Table 1 shows the mean change in microhardness values of tested restorative materials after immersion in various media.

**Graph 1:**
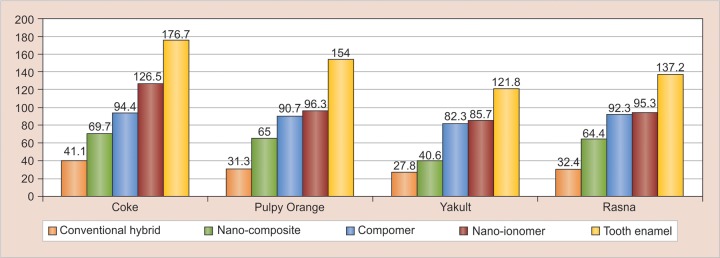
Change in microhardness for different restorative materials by one-way ANOVA test

**Graph 2:**
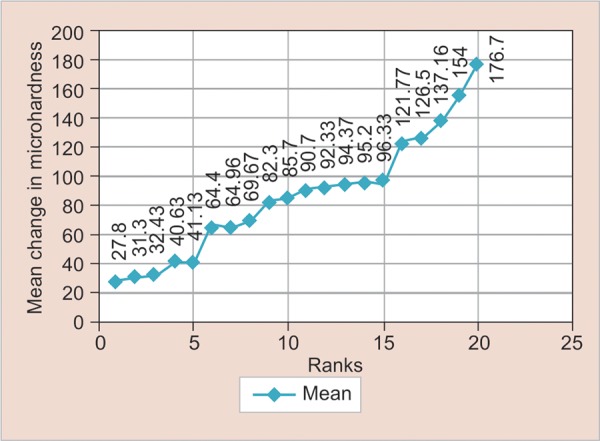
Ranking of various combinations of materials and beverages

One-way ANOVA test showed a significant change in microhardness in various immersion media for different restorative materials (Graph 1). The Tukey *post hoc* test showed significant difference in microhardness change between coke and Yakult for conventional hybrid composite (p = 0.03), for nano-ionomer (p = 0.02) and enamel (p = 0.04). The average surface hardness of the materials stored in Coca-Cola was different from that measured for Rasna and probiotic milk with exception of nano-composite and compomer groups. The overall ranking of combination of experimental drinks, restorative materials, and control shows that deciduous tooth enamel in Coca-Cola shows highest change in microhardness, whereas conventional composite in Yakult shows lowest change (Graph 2 and Table 2).

## DISCUSSION

When selecting the beverage, it should be borne in mind that the dissolution of enamel with erosion depends on the pH, the buffer capacity, length of exposure to the acid, and the temperature, as well as the concentrations of calcium, fluorine, and phosphate around the fluid.^12^

Damage to the enamel can occur when the pH drops below 5.5,^13^ which is referred to as critical pH. In the present study, Coca-Cola was found to be the most acidic drink with pH of 1.86, followed by Pulpy Orange, 3.43; Rasna 3.46; and the highest pH was shown by Yakult, 3.69. Thus, all the tested drinks had a pH range from 1.86 to 3.69, which is well below the critical pH. This was quite similar to the finding of Barac et al^14^ who showed that the erosion of the enamel surfaces occurred when exposed to Coca-Cola, orange juice, Cedevita, and Guarana. Similar pH values were obtained for coke, lemonades, and fermented milk as reported by de Mesquita-Guimaraes et al,^15^ Owens et al,^16^ Lodi et al,^17^ and Fatima et al.^5^ Coca-Cola was chosen for the study, as it is one of the oldest brands in the market with highest consumption and lowest pH. As lifestyles have changed through the decades, the growing number of consumers striving for a healthier diet has increased the consumption of natural juice and fruits. Oranges are particularly significant, as they are often consumed by children and teenagers.^18^ Thus, Minute Maid Pulpy Orange was chosen, as it does not contain any preservatives and mimics fresh orange juice. Rasna Nimbu Pani was taken, as it is the most popular brand among children and largest manufacturer of preparatory drink in India.^19^

**Table 2:** **Table 2:** Ranking of various combinations of materials and beverages

*Rank (R-Mean)*		*Subgroups*		*Mean (change in microhardness)*	
1		Subgroup A (III)—conventional composite + Yakult		27.80	
2		Subgroup A (II)—conventional composite + Pulpy		31.30	
3		Subgroup A (IV)—conventional composite + Rasna		32.43	
4		Subgroup B (III)—nanocomposite + Yakult		40.63	
5		Subgroup A (I)—conventional composite + Coca-Cola		41.13	
6		Subgroup B (IV)—nanocomposite + Rasna		64.40	
7		Subgroup B (II)—nanocomposite + Pulpy		64.96	
8		Subgroup B (I)—nanocomposite + Coca-Cola		69.67	
9		Subgroup C (III)—compomer + Yakult		82.3	
10		Subgroup D (III)—nano-ionomer + Yakult		85.7	
11		Subgroup C (II)—compomer + Pulpy		90.7	
12		Subgroup C (IV)—compomer + Rasna		92.33	
13		Subgroup C (I)—compomer + Coca-Cola		94.37	
14		Subgroup D (IV)—nano-ionomer + Rasna		95.2	
15		Subgroup D (II)—nano-ionomer + Pulpy		96.33	
16		Subgroup E (III)—enamel + Yakult		121.77	
17		Subgroup D (I)—nano-ionomer + Coca-Cola		126.5	
18		Subgroup E (IV)—enamel + Rasna		137.16	
19		Subgroup E (II)—enamel + Pulpy Orange		154	
20		Subgroup E (I)—enamel + Coca-Cola		176.72	

To measure the length of indentations correctly, the surface of the enamel had to be polished flat so that indentations became symmetrical. Grinding or flattening removes a certain amount of enamel which can become more sensitive to acidic solutions, and irregularities which develop on the sample surface do not have to be a consequence only of erosion but also of grinding.^14^

The results suggested that conventional composite when immersed in various study drinks showed least change in microhardness (VHN) as compared with other restorative materials. A significant difference (p = 0.03) was found in change in microhardness when conventional hybrid was immersed in Coca-Cola (41.3) as compared with Yakult (27.8). Nanohybrid composite and compomer, when immersed in Coca-Cola, had higher decrease in microhardness with a mean difference of 69.67 and 94.36 respectively, although the difference between other drinks was not significant. There was significant difference of change in microhardness for nano-ionomer (p = 0.024) and tooth enamel (p = 0.049) when immersed in Coca-Cola as compared with Yakult. Enamel slabs showed highest change in microhardness with all the tested beverages.

This change in microhardness was due to:

- Curing light generally cures the macrofil and heavy filled hybrids better than other restoratives, thus affecting the surface hardness.^20^
- When in contact with acid, there is a reported loss of structural ions from glass phase of polyacid-modified composites, thus reducing the microhardness.^6^
- Acid attack is reported to be positively related to the type of acid and the pH of the drink^5^ and thus Coca-Cola had highest erosive effect on enamel.^21^ As pH decreases from 6 to 3, a decrease in hardness is by factor of 5.^6^
- Prolonged time^22^ and frequent contact^15^ between the beverages and enamel and restorative material increase the opportunity for erosion to occur.
- It is found that fermented milk has fluoride, calcium, and phosphate, which adds a protective effect to the solution^17^ and thus in the present study, Yakult showed least potential for erosion.

The results of the present study were in accordance with other studies. Ren^23^ reported 85% reduction in surface enamel hardness, indicating an almost complete loss of minerals on the surface layer following immersion with orange juice as compared with whitening and polishing agents. de Melo et al^13^ found that processed and freshly squeezed orange juices were erosive to enamel. Taher^24^ reported similar results to that of the present study and found that hybrid composites had higher surface hardness as compared with nanocomposites, nanohybrids, and nano-ionomer. Lodi et al^17^ and de Mesquita-Guimaraes et al^15^ reported that fermented milk did not promote erosion of dental enamel. Also, Yamamoto et al^12^ reported that Coca-Cola soft drink had the greatest erosive effects on enamel when compared with other tested drinks. In accordance with the present study, Fatima et al^5^ showed that most acidic drinks had greatest erosive effect on enamel. Goyel et al^25^ reported that erosion of enamel was significantly higher than tooth-colored restorations.

Similar results were reported by other studies.^5,10,26,27^ Thus, conventional hybrid can be used efficiently as the effect of acidic beverages on the microhardness of this material is relatively lesser, although other factors should also be kept in mind.

## CONCLUSION

Within the limitations of the current study, the following conclusions were drawn:

- Coca-Cola showed lowest pH followed by Pulpy Orange, Rasna, and Yakult when the pH of various drinks was compared. Also, there was direct relation of pH with erosive potential of drinks. Maximum erosion was by immersion of restorative materials and tooth enamel in Coca-Cola and least by immersion in Yakult.
- When comparing the effects of common drinks on the surface hardness of different restorative materials and tooth enamel, the change in surface microhard-ness was found to be higher when specimens were immersed in Coca-Cola as compared with Pulpy Orange, Rasna, and Yakult.
